# Thermal Decomposition Behavior of Hydroxytyrosol (HT) in Nitrogen Atmosphere Based on TG-FTIR Methods

**DOI:** 10.3390/molecules23020404

**Published:** 2018-02-13

**Authors:** Jun-Ling Tu, Jiao-Jiao Yuan

**Affiliations:** Department of Chemical Engineering , School of Chemical Engineering and Energy Technology, Dongguan University of Technology, Dongguan 523808, China; tujl@dgut.edu.cn

**Keywords:** hydroxytyrosol, thermal decomposition, kinetics, TG-FTIR method

## Abstract

The thermal decomposition behavior of olive hydroxytyrosol (HT) was first studied using thermogravimetry (TG). Cracked chemical bond and evolved gas analysis during the thermal decomposition process of HT were also investigated using thermogravimetry coupled with infrared spectroscopy (TG-FTIR). Thermogravimetry-Differential thermogravimetry (TG-DTG) curves revealed that the thermal decomposition of HT began at 262.8 °C and ended at 409.7 °C with a main mass loss. It was demonstrated that a high heating rate (over 20 K·min^−1^) restrained the thermal decomposition of HT, resulting in an obvious thermal hysteresis. Furthermore, a thermal decomposition kinetics investigation of HT indicated that the non-isothermal decomposition mechanism was one-dimensional diffusion (D1), integral form *g*(*x*) = *x*^2^, and differential form *f*(*x*) = 1/(2*x*). The four combined approaches were employed to calculate the activation energy (*E* = 128.50 kJ·mol^−1^) and Arrhenius preexponential factor (ln *A* = 24.39 min^−1^). In addition, a tentative mechanism of HT thermal decomposition was further developed. The results provide a theoretical reference for the potential thermal stability of HT.

## 1. Introduction

Hydroxytyrosol (HT), 3,4-dihydroxy phenethyl alcohol, is an important active compound in olive oil and olive leaf [1,2]. Small-molecule HT contains three free hydroxyl groups and presents strong bioactivity, such as antioxidant [3,4], antimicrobial [5,6], and anti-inflammatory properties [7], in addition to antitumor activity to inhibit the proliferation of human promyelocytic leukemia HL60 cells [8], human colon cancer HT-29 cells [9], and MCF-7 human breast cancer cells [10]. Many works have reported on the HT chemical structure and derivatives [11,12], antitumor activity and mechanism [13], and pharmacokinetics [14] for anticancer drugs or cancer adjuvant therapy. Therefore, HT or HT additives could be used as potentially safe natural medicine. 

As reported by Luo et al. [15], HT has a strong biological instability on account of its multiple hydroxyl groups structure. This greatly affects the relevant preparations quality and clinical application effect due to low bioavailability. In addition, drug thermal stability plays a crucial role in storage, quality control, and validity period prediction. Thermal analysis is a promising technology that has been used to investigate the thermal stability and decomposition kinetics of drug compounds [16,17]. Kinetic analysis has attracted great attention in the modern history of thermal decomposition studies [18,19,20,21]. Practical application mainly emphasizes the prediction of process rates and material lifetime. Theoretical application is the description of three kinetic factors that are determined by experimental investigation, which can be used to describe the thermodynamic properties. Material weight dynamic change is obtained with the temperature change by thermogravimetry (TG), and the cracked simulation of chemical bond is analyzed by TG-FTIR to determine thermal decomposition characteristics and decomposition kinetic mechanism. Combined with assistive techniques, thermal analysis depicts the complete thermal behavior of a drug. Thus, it is important to study the thermal decomposition kinetics of HT for new antitumor drug thermal stability. However, kinetic research of the HT decomposition process has rarely been reported in the literature.

The aim of this work is to analyze the kinetics of HT thermal decomposition and its cracked chemical bond mechanism in the process of TG and TG-FTIR. At the same time, multiple heating rate methods (Kissinger, Flynn-Wall-Ozawa (F-W-O), Friedman methods) and a single heating rate method (Coats-Redfern method) were used to determine the kinetic parameters, such as activation energy and preexponential factor, and to propose the most probable reaction mechanism of HT decomposition and its kinetic model.

## 2. Results and Discussion

### 2.1. Thermal Decomposition Behavior

**Figure 1:**
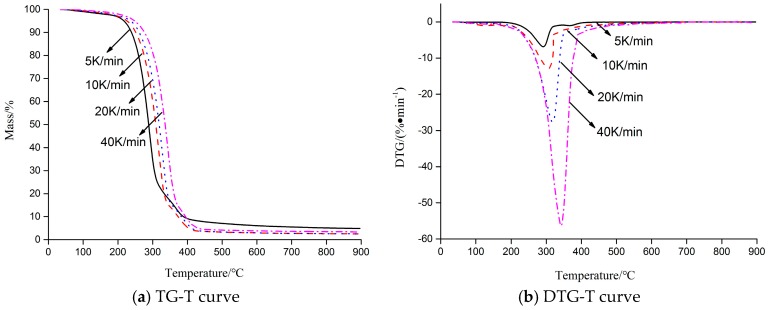
TG-DTG curve of hydroxytyrosol (HT) at different heating rates. (TG: Thermogravimetry; DTG: Differential thermogravimetry).

The thermal decomposition process of HT was carried out, and TG-DTG curves of HT at different heating rates are presented in Figure 1. As shown in Figure 1 (10 K·min^−1^), HT was stable and had almost no mass loss before 262.8 °C. After 262.8 °C, HT started to decompose quickly. At about 409.7 °C, HT decomposition was almost completed, and 2.68% solid char residue was observed. The TG curve showed that the decomposition of HT was completed in one step, and the temperature from 262.8 °C to 409.7 °C was main thermal decomposition stage. The DTG curve showed a sharp peak at 305.2 °C, the max loss ratio was −12.91%·min^−1^, and the weight loss was up to 54.32%.

In addition, the trend of TG-DTG curves of different heating rates was basically the same. As the heating rate increased, the beginning temperature of HT thermal decomposition also increased, as shown in Table 1. Thermal hysteresis became more and more evident and a high heating rate (over 20 K·min^−1^) could restrain the thermal decomposition of HT. Meanwhile, the negative peak of the DTG curve moved to a higher temperature zone, in which no pattern changes appeared. From the kinetic point of view, thermal behavior suggested that the thermal decomposition mechanism is independent of the heating rates and the reaction rate is a function of the temperature under the experimental conditions used in this study [22]. If the heating rate was too high, it resulted in the information loss of some intermediate products in the detected spectrum. If the heating rate was too low, it might result in a discrete TG spectrum.

### 2.2. Nonisothermal Kinetics of HT

The non-isothermal decomposition kinetics of HT was investigated using F-W-O, Friedman, Kissinger, and Coats-Redfern methods. The activation energy (*E*) and ln *A* calculated by the Kissinger method were 109.26 kJ·mol^−1^ and 21.82 min^−1^, respectively. The linear correlation coefficient (*R*^2^) was 0.9424. According to Friedman and F-W-O methods, the parameters ($\frac{dx}{dt}$ and temperature) of the HT thermal decomposition process were investigated at different conversion ratios, and the related kinetic parameters are presented in Table 2. The results inferred that the correlation coefficients (*R*^2^) were all good. According to kinetic data, a new increase in the *E* value was observed, which suggested that the decomposition became more difficult as conversion ratio rose, so the mean activation energy was needed in the process. As calculated, the mean *E* value was 143.1 and 128.61 kJ·mol^−1^, respectively. From Table 2, it was indicated that the *E* value calculated by the Friedman method was slightly higher than that calculated by the F-W-O method. Moreover, *E* was lower when determined with the Kissinger method, compared with the Friedman and F-W-O methods. These differences were related to the used form by the kinetic methods, which considered that the temperature and *E* did not both depend on the conversion degree [23,24]. Therefore, significant differences among *E* values determined by the differential and integral reaction models often existed.

The kinetic parameters obtained by the Coats-Redfern method are presented in Table 3. From Table 3, we can see that *E* varied with the rise of heating rates for the same reaction model [20]. Furthermore, the *E* value increased when the heating rate increased from 5 to 40 K·min^−1^. The reason for this phenomenon might be that as the heating rate became higher, it became harder to reach the needed temperature in the short time period, resulting in more energy being required to decompose HT thoroughly. Moreover, it was demonstrated that the linear dependence of fitting results obtained by models were better except F2, F3, and P4 models, but the difference between *E* and *A* values acquired by these models were obvious. It was shown that several kinds of mathematical models matched with the same set of data. Therefore, good linear dependence could not determine the rationality of the selected mathematical reaction model. So, in this study, Kissinger, Friedman, and F-W-O methods were selected to verify *E* and the mathematical reaction model, which calculated using the Coats-Redfern method. The range of *E* values achieved by the three abovementioned methods was 109.26–143.1 kJ·mol^−1^, and only the *E* value calculated by model D1 was closest to this result, being in the range of 114.36–137.18 kJ·mol^−1^. Therefore, it was concluded that the D1 model (one-dimensional diffusion) could exactly describe the thermal decomposition process of HT, and the forms of the integral and differential equations for the mathematical reaction model were *g*(*x*) = *x*^2^ and *f*(*x*) = 1/(2*x*), respectively.

**Figure 2:**
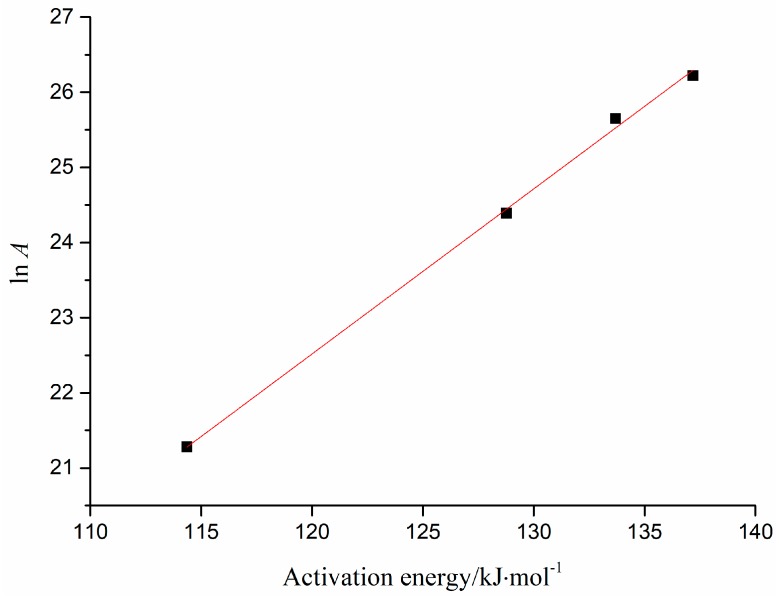
Kinetics compensation effect of HT thermal decomposition by different heating rates.

The kinetics compensation effect of HT thermal decomposition by different heating rates is presented in Figure 2. The linear equation (ln *A* = 0.2197*E* − 3.8404) was calculated by fitting the data of *E* and ln *A* from D1 model. Thereby, *E*_0_ was 128.5 kJ·mol^−1^, which was calculated by the mean *E* in model D1, and ln *A* was 24.39 min^−1^, according to the above linear equation calculation. The differential form *f*(*x*) = 1/(2*x*), and *E* and *A* of HT were substituted into the original kinetics, thus a kinetic expression of the HT thermal decomposition process was achieved, and the specific expression was: $\frac{dx}{dT}=\frac{3.92\times 10^{10}}{\beta }\exp(-\frac{129000}{8.314\times T})\frac{1}{2x}$.

### 2.3. Chemical Bond Change by TG-FTIR

**Figure 3:**
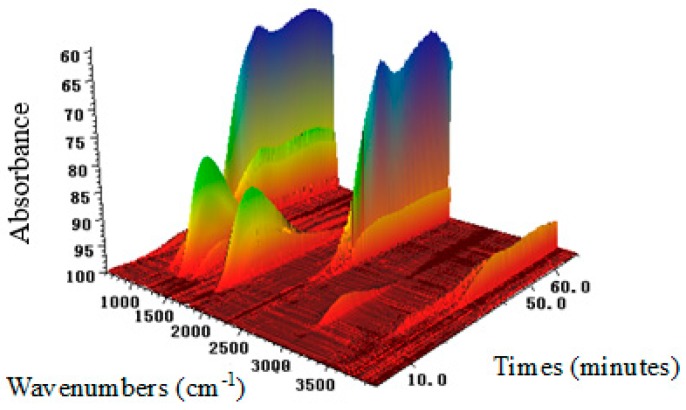
Three-dimensional infrared spectra of HT.

The HT volatiles gasified during thermal decomposition were measured using TG-FTIR, and the three-dimensional (3D) FTIR analysis is presented in Figure 3. The change of spectral intensity along the time was similar to the TG results. However, the temperature at the spectral intensity peak was determined by TG because of the time delay from TG to FTIR [19]. Absorbance information at different wave numbers and time could be achieved from the 3D FTIR of HT. It can be seen that most of the substances were evolved between 20 and 40 min.

**Figure 4:**
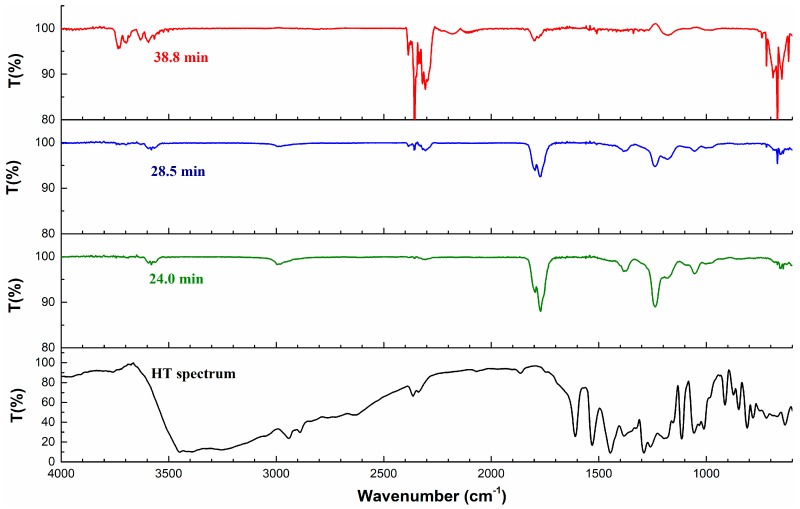
Infrared spectra of HT at particular time points.

The infrared spectra of HT and evolved gas from the HT thermal decomposition are presented at specific time points in Figure 4 and Table 4, respectively. The spectra at 24.0 min (starting time point of the decomposition), 28.5 min (time point at which the maximum weight loss rate was reached), and 38.8 min (time point at completion of the decomposition) were selected to analyze the variation of the functional groups and evolved gas with wavelengths from HT thermal decomposition. The HT spectra were the infrared spectra of HT before TG. It should be noted that the maximum absorbance at 28.5 min was in accordance our expectations, considering the DTG curve described in Figure 1.

Because of the hydroxyl groups, the HT spectrum exhibits a broad peak at 3500 cm^−1^ in Figure 4. When the decomposition reached 24.0 min, signals were observed between 1850–1700 cm^−1^ and 1400–1000 cm^−1^, which then disappeared till 38.8 min. In addition, signals were also observed between 3750 and 3500 cm^−1^, which were basically the characteristic absorption peaks of water. By this token, water was produced first. When the decomposition reached 28.5 min, the C–H stretching, C–O stretching, and =C–C=C– (aromatic ring) was observed at 3000, 1230, and 1600 cm^−1^, and it was demonstrated that the signal peak of phenol existed. When the analysis reached 38.8 min, the signals at 3000 cm^−1^ and 1750 cm^−1^ disappeared, and it was shown that the middle product phenol was further decomposed. In addition, signals were observed between 2300 cm^−1^ and 700–600 cm^−1^, which appeared at 28.5 min and rose until 38.8 min. A literature survey indicated that the wavenumber between 2300 cm^−1^ and 700–600 cm^−1^ was the characteristic absorption peak of CO_2_. It was noted that CO_2_ was gradually generated in the interval between 28.5 and 38.8 min. The presence of H_2_O and CO_2_ could be identified by GC after complete decomposition. Of course, particular chemical bonds or functional groups were also produced in the decomposition, and the evolved substances mainly included H_2_O and CO_2_.

### 2.4. Proposed Mechanism of HT Thermal Decomposition

As shown in Table 4, the absorbance of saturated –CH_3_, –C=C–, –OH, –C–O revealed that a side chain of HT (–CH_2_CH_2_OH) might have cracked from aromatic ring at 24.0 min, and substances with –OH might be primary alcohol or phenols. With the absorbance of these chemical bonds constantly decreasing along the time direction during the thermal decomposition process, CO_2_ and H_2_O production increased constantly. It was showed that –C–C–, –C=C–, and –OH continually were used to reengineer the structure and further form CO_2_, H_2_O, and carbonaceous material. This may be related to the activation energy change in the thermal decomposition of HT, which could affect HT stability. Different chemical bonds would need different levels of fracture energy. With the increase of temperature, energy was supplied to the thermal decomposition, leading to the occurrence of the abovementioned phenomenon.

**Figure 5:**
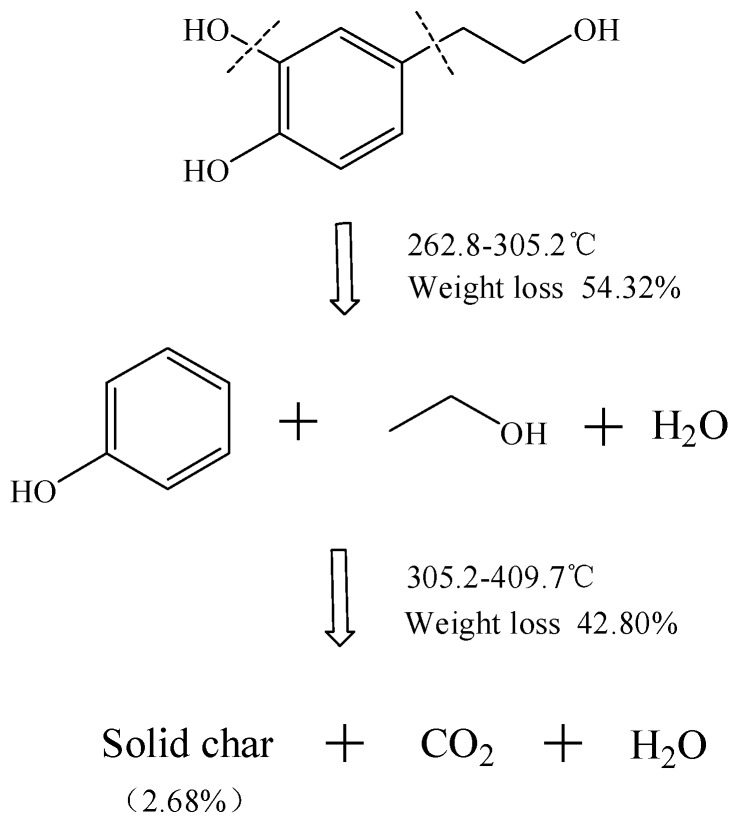
Proposed mechanism of HT thermal decomposition under a N_2_ atmosphere.

To summarize, internal chemical bonds of HT were ruptured to produce small molecules and volatile compounds detected during the thermal decomposition processes, completing the main decomposition of HT. The tentative thermal decomposition hypothesis is proposed in Figure 5. A rupture route is presented, and it is shown that HT was decomposed at 262.8 °C. Due to multiple hydroxy groups, it produced water in the beginning. The mass loss was 54.52% in the interval between temperatures 262.8 and 305.2 °C, which suggested the loss of phenol (theoretical calculation = 61.04%), and CO_2_ was generated after a series of chemical recombinations. At 409.7 °C, decomposition was completed and a solid residue of 2.68% was observed, which was related to carbonaceous material. Therefore, the final products were CO_2_, water, and 2.68% solid char.

## 3. Materials and Methods

### 3.1. Material

HT standard (purity >98%, molecular weight 154) was purchased from Sigma (St. Louis, MO, USA). 

### 3.2. Methods

The thermal decomposition process of HT was carried out with a thermogravimetry analyzer (NETZSCH TG 449C, NETZSCH, Selb, Germany) coupled with an FTIR spectrophotometer (Bruker Tensor 27 FTIR, Berlin, Germany). The TG analysis were completed at different heating rates (5, 10, 20, 40 K min^−1^) from 313 to 1173 K under a nitrogen atmosphere (35 mL·min^−1^), using about 10 mg HT, respectively. The FTIR instrument was connected to the TG analyzer by a pipe and a flow cell, which were preheated to 453 K to prevent condensation of the evolved gases. According to the FTIR real-time tracking on the pyrolysis gases, the spectra of some compounds during decomposition could be recorded in the scanning range from 4000 to 400 cm^−1^.

### 3.3. Theoretical Analysis

*k*(*T*) stands for the temperature-dependent rate constant, which can be described by the Arrhenius Equation (1):

(1) $$k(T)=Ae^{-E/RT}$$

#### 3.3.1. Kissinger Method

The Kissinger method [25] is expressed by Equation (2):

(2) $$\ln(\frac{T_{P}^{2}}{\beta })=\ln(\frac{E}{R})-\ln A+\frac{E}{RT_{P}}$$

By plotting ln (*β*/*T_p_*^2^) versus 1/*T_p_*, *E* and *A* can be calculated based on the slope (−*E*/R) and intercept ln(*AR*/*E*), respectively.

#### 3.3.2. Flynn-Wall-Ozawa Method

This method [26] is an integral method, by which the activation energy *E* can be obtained via the conversion of the reactant. The integral form of the Flynn-Wall-Ozawa (F-W-O) method is represented by Equation (3):

(3) $$\ln\beta =\ln(\frac{AE}{R})-\ln g(x)+5.3305-1.052\frac{E}{RT}$$

Definitions of all of the variables and parameters are the same as those described for previous equations. Since the value of ln (*AE*/*R g*(*x*)) is approximately constant when the values of *x* are the same at different heating rates *β*, the plot ln *β* versus 1/*T* is approximately linear. Thus, by plotting ln *β* against 1/*T* at certain conversion *x*, the slope (−1.052 *E*/*R*) can be used to calculate *E*.

#### 3.3.3. Friedman Method

The Friedman method [27] is expressed by Equation (4):

(4) $$\ln(\frac{dx}{dt})=\ln[Af(x)]-\frac{E}{RT}$$

Definitions of all of the variables and parameters are the same as those described for previous equations. By plotting ln(d*x*/d*t*) versus 1/*T*, *E* can be calculated based on the slope (−*E*/R).

#### 3.3.4. Most Probable Reaction Mechanism and Mathematical Model

Many mathematical reaction models may show a good linear relationship, resulting in a huge difference of reactive kinetic parameters to the same substance. Obviously, this is caused by the difference between the selected reaction mathematical models and the actual kinetic process, so the appropriate selection of the reaction model can influence the accuracy of the HT thermal decomposition mechanism. To choose the most probable mathematical reaction model of HT, 15 kinds of frequently used mathematical reaction models (see Table 5) were substituted into the Coats-Redfern equation to determine *E* [28], respectively. Then, the *E* value was selected to approach to the mean *E* calculated using the three abovementioned methods (Kissinger, F-W-O, and Friedman methods), and the corresponding mathematical model is the most probable reaction mathematical model of HT.

The Coats-Redfern equation [29] is expressed by Equation (5):

(5) $$\ln\frac{g(x)}{T^{2}}=\ln\frac{AR}{\beta E}-\frac{E}{RT}$$

By substituting *g*(*x*) in Table 5 into Equation (5) and plotting ln[*g*(*x*)/*T*^2^] versus 1/*T*, *E* and *A* of the different mathematical reaction models can be calculated based on the slope (−*E*/*R*) and intercept (ln(*AR*/*βE*)).

#### 3.3.5. Calculation of ln *A*

The preexponential factor is one of the important kinetic parameters of the reaction, which is the constant *A* in the Arrhenius Equation (1). Tt is determined by the reaction feature of the reactant and it is independent of temperature, so the calculation of *A* is important for understanding the reaction feature of HT. As compensation effect exists in *E* and *A*, Equation (6) is usually used to calculate ln *A*:

(6) lnA=aE+b

First, fitting *E* and ln *A* of different heating rates for the most probable mathematical reaction model, the values of *a* and *b* can be obtained from the slope and intercept of the fitted curve. Then, the average value of *E* of different heating rates for the most probable reaction mathematical model is substituted, and ln *A* can be determined by Equation (6).

## 4. Conclusions

TG-FTIR technology were used to analyze the HT thermal stability and decomposition kinetics mechanism, as well as a simulation of the occurrence of a cracked chemical bond. TG-DTG curves revealed that the main thermal decomposition of HT was completed in the temperature interval between 262.8 and 409.7 °C. The non-isothermal thermal decomposition mechanism of HT was a one-dimensional diffusion, with integral form (*g*(*x*) = *x*^2^) and differential form (*f*(*x*) = 1/(2*x*)). The four combined approaches were employed to calculate *E* = 128.50 kJ·mol^−1^ and ln *A* = 24.39 min^−1^. During the thermal decomposition process, –CH_3_, –C=C–, –OH, and –C–O were produced at a continuously accelerated rate, and CO_2_, H_2_O, and solid char were the end products after completion of the decomposition.

## Figures and Tables

**Table 1:** Pyrolysis characteristic parameters of HT.

Heating Rate (K·min^−1^)	Beginning Temperature (°C)	End Temperature (°C)	*T*_m_ (°C)	Maximum Loss Ratio (%·min^−1^)	Solid Char (%)
5	252.9	395.1	291.4	−6.85	4.85
10	262.8	409.7	305.2	−12.91	2.68
20	273.6	415.3	314.8	−27.71	2.53
40	298.7	418.9	341.9	−56.35	3.34

**Table 2:** Kinetic parameter of HT thermal decomposition by Friedman and Flynn-Wall-Ozawa (F-W-O) methods.

Conversion	Friedman Method		F-W-O Method	
	*E* (kJ·mol^−1^)	*R*^2^	*E* (kJ·mol^−1^)	*R*^2^
0.1	121.36	0.9329	125.71	0.9950
0.2	118.89	0.9744	118.41	0.9894
0.3	124.24	0.9903	118.56	0.9858
0.4	126.51	0.9815	119.64	0.9815
0.5	127.05	0.9673	120.19	0.9783
0.6	133.66	0.9317	123.37	0.9784
0.7	249.99	0.9	174.39	0.9503
Mean	143.1	—	128.61	—

“—” means without this value.

**Table 3:** Kinetic parameter of HT thermal decomposition by the Coats-Redfern method.

No.	*β* = 5 K·min^−1^			*β* = 10 K·min^−1^			*β* = 20 K·min^−1^			*β* = 40 K·min^−1^		
	*E*	ln *A*	*R*^2^	*E*	ln *A*	*R*^2^	*E*	ln *A*	*R*^2^	*E*	ln *A*	*R*^2^
F1	71.80	12.91	0.9782	79.79	14.90	0.9937	82.91	15.97	0.9934	85.04	16.56	0.9911
F2	96.65	18.86	0.987	104.83	20.97	0.9682	109.56	22.14	0.968	112.36	22.72	0.9654
F3	126.61	26.15	0.9779	136.33	28.15	0.9349	141.52	29.45	0.9348	145.14	29.99	0.9323
D1	114.36	21.28	0.9489	128.77	24.39	0.9976	133.69	25.65	0.9975	137.18	26.22	0.9961
D2	125.43	23.27	0.9616	140.43	26.43	0.9991	145.77	27.74	0.9989	149.57	28.30	0.9972
D3	138.74	24.97	0.9732	154.32	28.16	0.998	160.17	29.53	0.9978	164.33	30.08	0.9958
D4	129.83	22.83	0.9660	145.03	26.00	0.9991	150.54	27.33	0.9989	154.45	27.88	0.9971
A2	31.30	3.64	0.9707	35.21	5.03	0.9924	36.69	5.92	0.9921	37.62	6.55	0.9892
A3	17.80	0.27	0.959	20.35	1.47	0.9906	21.28	2.30	0.9902	21.81	2.93	0.9865
R1	52.58	8.11	0.9392	59.70	10.07	0.9971	62.08	11.05	0.997	63.69	11.66	0.9954
R2	61.49	9.66	0.9629	69.05	11.64	0.9988	71.77	12.66	0.9986	73.62	13.27	0.9965
R3	64.77	10.07	0.9689	72.48	12.06	0.9978	75.32	13.09	0.9976	77.26	13.82	0.9954
P2	21.69	1.04	0.9107	25.17	2.42	0.9956	26.27	3.27	0.9954	26.94	3.91	0.9933
P3	11.39	−1.67	0.8598	13.65	−0.45	0.9927	14.34	0.36	0.9926	14.69	1.01	0.9895
P4	6.24	−3.31	0.7605	7.90	−2.12	0.9867	8.37	−1.33	0.9868	8.57	−0.69	0.982

**Table 4:** FTIR analysis from HT thermal decomposition products.

Wavenumber (cm^−1^)	Vibration of Corresponding Bond	Functional Group
3964–3500, 1300–1800	O–H stretching	H_2_O
2313–2361, 669	C=O stretching, C=O bending	CO_2_
1684–1745, 2822–2915	C=O stretching, C–H stretching	R–CHO
1000–1300, 3585–3650	C–O stretching, O–H stretching	R–OH
2900–3000	C–H stretching	–CH_3_ (saturation)
3066	C–H stretching (aromatic ring)	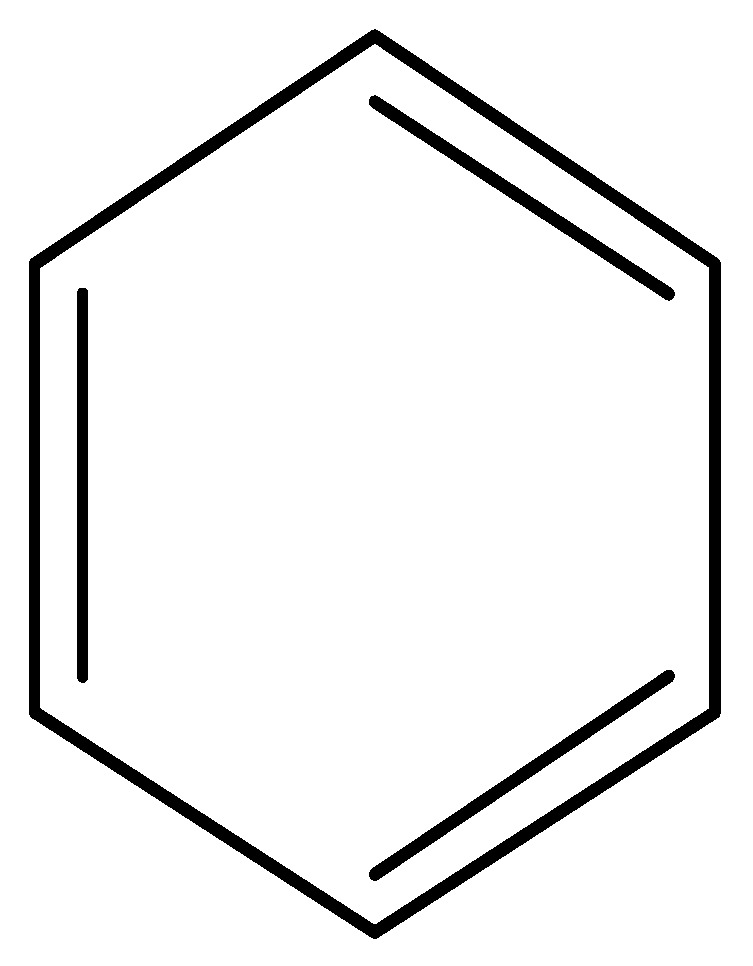
1600	=C–C=C– (aromatic ring)	
1715	C=C stretching	–C=C
3200–3650	O–H stretching	–OH
1000–1300	C–O stretching	–C–O

**Table 5:** Kinetic mechanism functions of pyrolysis.

Number	Model	Reaction Mechanism	*g*(*x*)
1 (F1)	Chemical reaction	*n* = 1	−ln(1 − *x*)
2 (F2)		*n* = 2	(1 − *x*)^−1^ − 1
3 (F3)		*n* = 3	[(1 − *x*)^−2^ − 1]/2
4 (D1)	Diffusion	One-dimensional diffusion	*x*^2^
5 (D2)		Two-dimensional diffusion	(1 − *x*)ln(1 − *x*) + *x*
6 (D3)		Three-dimensional diffusion (Jander equation)	[1 − (1 − x)1/3]2
7 (D4)		Three-dimensional diffusion (Ginstling-Brounshtein equation)	(1 − 2*x*/3) − (1 − *x*)^2/3^
8 (A2)	Random nucleation and growth	Two-dimensional	[−ln(1 − *x*)]^1/2^
9 (A3)		Three-dimensional	[−ln(1 − *x*)]^1/3^
10 (R1)	Interfacial reaction	One-dimensional	*x*
11 (R2)		Cylindrical symmetry	[1 − (1 − x)1/2]
12 (R3)		Spherical symmetry	[1 − (1 − x)1/3]
13 (P2)	Exponential nucleation	Power function, *n* = 1/2	*x*^1/2^
14 (P3)		Power function, *n* = 1/3	*x*^1/3^
15 (P4)		Power function, *n* = 1/4	*x*^1/4^

## Abbreviations

d*x*/d*t*	stands for a rate of conversion;
*x*	stands for the conversion of the reaction, which is defined as *x* =(*w*_0_ − *w_t_*)/(*w*_0_ − *w_f_*), where *w*_0_ and *w_f_* stand for the initial and final masses of the sample at each stage of decomposition (mg), respectively, and *w_t_* stands for the mass of the sample at time *t* (mg);
*f*(*x*)	stands for a differential mathematical model function of kinetics, which depends on the reaction type and mechanism;
*g*(*x*)	stands for an integral mechanism model function of kinetics, which depends on the reaction type and mechanism;
*k*(*T*)	stands for the temperature-dependent rate constant, which can be described by the Arrhenius equation;
*A*	stands for the preexponential factor (min^−1^);
*R*	stands for the gas constant 8.314 J·(mol·K)^−1^;
*E*	stands for the apparent activation energy (kJ·mol^−1^);
*T*	stands for the absolute temperature (K);
*Β*	stands for the heating rate (K·min^−1^);
*T_p_*	stands for the absolute thermodynamic temperature at which the weight loss rate reaches a maximum value (K).
